# Expression analysis of *centrin* gene in promastigote and amastigote forms of *leishmania infantum* iranian isolates: a promising target for live attenuated vaccine development against canine leishmaniasis

**DOI:** 10.1186/s12917-021-02816-x

**Published:** 2021-04-14

**Authors:** Mohammad Javad Abbaszadeh Afshar, Samira Elikaee, Reza Saberi, Sina Mohtasebi, Mehdi Mohebali

**Affiliations:** 1 Department of Medical Parasitology and Mycology, School of Public Health, Tehran University of Medical Sciences, Tehran, Iran; 2 School of Pharmacy, Pacific University, Hillsboro, Oregon USA; 3 Department of Medical Parasitology and Mycology, School of Medicine, Mazandaran University of Medical Sciences, Mazandaran, Iran; 4 Center for Research of Endemic Parasites of Iran (CREPI), Tehran University of Medical Sciences, Tehran, Iran

**Keywords:** *Centrin* gene, *Leishmania infantum*, Canine leishmaniasis, Gene expression, Real‐time RT-PCR

## Abstract

**Background:**

*Leishmania* parasites express various essential proteins in different growth phases (logarithmic/stationary) and forms (promastigote/amastigote). Targeting the genes encoding such proteins paves the way for controlling these parasites. *Centrin* is an essential gene, which its protein product seems to be vital for the proliferation of *Leishmania* parasites. Herein, this study was contrived to analyze the expression level of the *centrin* gene in different growth phases and forms of *Leishmania infantum* (*L. infantum*) parasites isolated from various endemic areas of canine leishmaniasis (CanL) in Iran.

**Results:**

All three collected isolates were identified as *L. infantum* using polymerase chain reaction-restriction fragment length polymorphism (PCR-RFLP). Real-time reverse transcription (RT)-PCR revealed a statistically significant up-regulation (3.13-fold) in the logarithmic phase promastigotes compared to stationary ones (*p* < 0.01), whereas *centrin* was expressed equally in intracellular amastigotes at different time points during cell culture. Also, our finding revealed a slight up-regulation of the *centrin* gene (1.22-fold) in the intracellular amastigotes compared to logarithmic phase promastigotes, which was found statistically non-significant (*p* > 0.05).

**Conclusions:**

*Centrin* gene in Iranian isolates of *L. infantum* is more expressed in exponential than stationary phases and seems to be considered as a promising target in the development of a genetically modified live attenuated vaccine for CanL control.

## Background

Canine leishmaniasis (CanL) caused by *Leishmania infantum* (*L. infantum*), as a challenging global zoonosis, is endemic in some areas in Iran and 70 countries around the world [1–3]. The parasite is transmitted accidentally from the infected dogs, as the main reservoir of CanL, to humans through sandflies biting and cause visceral leishmaniasis (VL), the most severe clinical form of leishmaniases. However, the number of new symptomatic human VL cases reported annually is around 100 cases in Iran, reviewing the conducted studies on domestic dogs indicates the disease is prevalent at least in half of the country’s provinces [4, 5]. Therefore the formation of the new VL foci is not far-fetched in Iran.

Vaccination is the most cost-effective control strategy for controlling CanL, in that successful immunization of dogs could interrupt transmission and reduce the incidence of VL significantly [6]. Several approaches for vaccine development against CanL have been evaluated such as killed parasites, live attenuated parasites, recombinant proteins, and naked DNA [6]. Unlike other approaches, live attenuated vaccines lead to interactions with the host immune system resulting in a wide range of antigens similar to natural parasite infection, hence could provide more protection [7]. Development of live attenuated *Leishmania* parasites, including long-term in vitro cultures, chemical mutagenesis, and irradiation, is associated with some problems like undefined random genetic mutations or potential reversion virulence. While, genetically modified live attenuated vaccines, through targeting the genes encoding essential proteins like growth-regulating or virulence genes, are safe [8]. To target such genes, it would be essential to analyze their function in different forms and growing phases of *Leishmania* parasites.

Various qualitative and quantitative biochemical parameters affect the differentiation, proliferation, pathogenesis, and survival of *Leishmania* parasites, which are different in each form and growing phase of the parasite [9–11]. For instance, the genes related to infectivity are up-regulated in metacyclic promastigotes [11]. Amastigotes also change their gene expression levels to survive and multiply in the hostile environment inside the macrophage’s phagolysosomes. Some of these modifications in gene expression levels may point to unique parasite genes that could be targeted to the development of a prophylactic vaccine [12, 13].

Several studies have characterized genes that may have an essential role in the growth and proliferation of the *Leishmania* parasite [10, 14]. *Centrin* gene and its function concerning the *Leishmania* parasite proliferation have been described as one of the mentioned genes [15]. *Centrin* gene product is one of the several regulatory proteins required for duplication or segregation of the centrosome in higher eukaryotes and basal bodies in lower eukaryotes [16]. The role of *centrin* in cytokinesis of yeast and HeLa cells and also the formation of flagellate in *Chlamydomonas* has been documented in several studies [17–20]. Also, *centrin* is described as one of the essential factors in cell division of several protozoa such as *Leishmania*, *Trypanosoma*, and *Plasmodium* [15, 21, 22].

To target *centrin* for developing a genetically modified live attenuated vaccine against CanL in Iran, it would be essential to analyze its expression in the different forms and growing phases of *L. infantum* parasites. Hence, this study aimed to compare the expression level of the *centrin* gene in the logarithmic phase compared to the stationary phase of *L. infantum* promastigotes and also the comparison of the expression level of the *centrin* gene in the promastigote and amastigote forms of *L. infantum* Iranian isolates using real-time reverse transcription (RT)-PCR.

## Results

### Molecular characterization

**Fig. 1:**
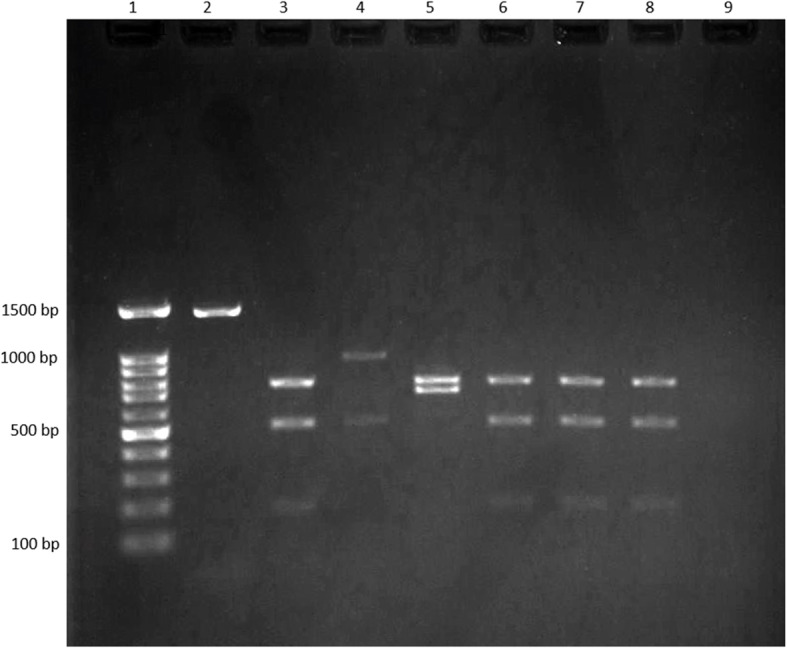
Digestion pattern of NAGT amplicon using the Acc1 enzyme in the *Leishmania* isolates. **Lane 1**: molecular weight marker (100 bp), **Lane 2**: undigested NAGT amplicon, **Lane 3–5**: positive controls (*L. infantum*, *L. major*, and *L. tropica*, respectively), **Lane 6–8**: *L. infantum* isolates, **Lane 9**: none template control

All three *Leishmania* isolates collected from various endemic areas of Iran were identified as *L. infantum* using polymerase chain reaction-restriction fragment length polymorphism (PCR-RFLP). Following the N-acetylglucosamine-1-phosphate transferase (NAGT) gene digestion with the Acetyl-coenzyme A carboxylase 1 (Acc1) enzyme, all isolates exhibited three bands (780, 500, and 180 bp) on the agarose gel, which corresponds to *L. infantum* (Fig. 1).

### Complementary DNA (cDNA) synthesis and real‐time RT-PCR analysis

**Fig. 2:**
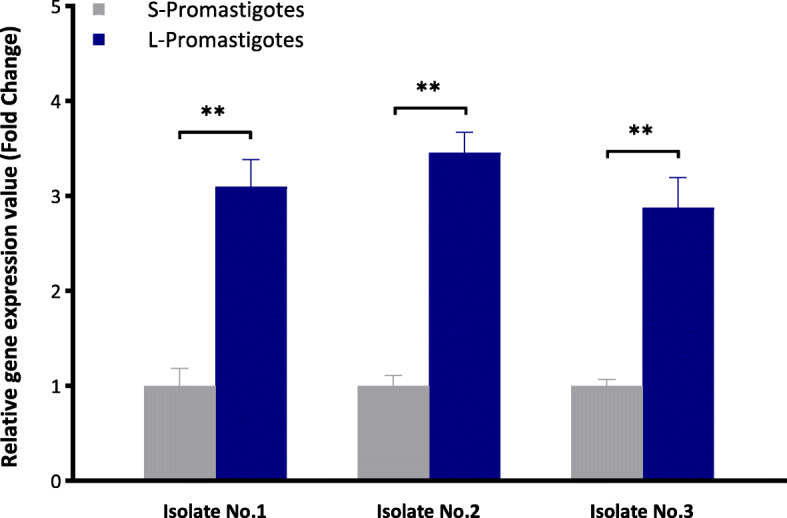
Relative expression level of the *centrin* gene in the stationary and logarithmic-phase *L. infantum* isolates, using the 2^−ΔΔct^ method. The expression of *α-tubulin* was used to normalize the data. The values are the mean ± SD of two independent experiments. S: stationary-phase, L: logarithmic-phase, **: *p* < 0.01

The integrity of synthesized cDNA was determined by conventional PCR through *α-tubulin* amplification as a housekeeping gene. Real-time RT-PCR was used to compare the relative expression of the *centrin* gene in the logarithmic and stationary phase promastigotes and also in the logarithmic promastigote and amastigote forms of *L. infantum* isolates. Figure 2 shows significant up-regulation of the *centrin* gene in the logarithmic phase compared to stationary phase *L. infantum* promastigotes in all three collected isolates. The overall average mRNA expression of the *centrin* gene in the logarithmic phase promastigotes was 3.13-fold of its expression in stationary phase ones (*p* < 0.01).

**Fig. 3:**
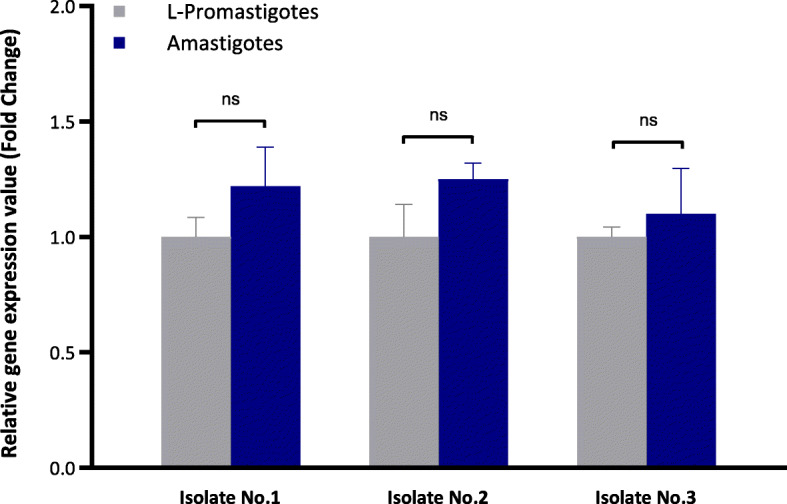
Relative expression level of the *centrin* gene in logarithmic-phase promastigote and amastigote forms of *L. infantum* isolates, using the 2^−ΔΔct^ method. The expression of *α-tubulin* was used to normalize the data. The values are the mean ± SD of two independent experiments. L: logarithmic-phase, ns: non-significant (*p* > 0.05)

The results of the current study also showed *centrin* expressed equally in the intracellular amastigotes at different time points (24 h and 72 h post-infection) during the cell culture (data not shown). Also, our results revealed a slight up-regulation of the *centrin* gene (1.22-fold) in the intracellular amastigotes compared to logarithmic phase promastigotes, which was found statistically non-significant (*p* > 0.05) (Fig. 3). Moreover, we investigated the *centrin* gene expression level among each *L. infantum* isolate, in which there was no significant difference (*p* > 0.05).

## Discussion

*Centrin* gene product is a cytoskeletal, calcium-binding protein located at the basal body region of *Leishmania* parasites. The nature of the basal body and the genes associated with this vital unit in *Leishmania* parasites are still poorly known. Centrin protein, besides calmodulin, γ-tubulin, and other types of centrin, is an essential regulatory protein required for duplicating the basal body in *Leishmania* parasites [15]. To investigate the expression pattern of the *centrin* gene in different growth phases (logarithmic/stationary) and forms (promastigote/amastigote) of *L. infantum*, isolated from various endemic areas of Iran, we conducted a real-time RT-PCR.

As our results revealed, the *centrin* gene expression pattern had no significant difference in *L. infantum* isolates of various geographical areas of Iran. Our findings showed a significant up-regulation of the *centrin* mRNA expression in the logarithmic phase compared to the stationary phase of *L. infantum* promastigotes in all three collected isolates. The overexpression of this protein in logarithmic phase *L. infantum* parasites confirms the finding of the study carried out on *Leishmania donovani* (*L. donovani*) parasites [23]. Based on the mentioned study, *Leishmania* centrin protein expression was high during the exponential growth phase of the parasite in culture and significantly declined in the stationary phase. This expression pattern suggests that *Leishmania centrin* may have an essential role in the proliferation of the parasite. Furthermore, since stationary promastigotes compare to logarithmic ones are highly motile [24], the specified expression pattern in the current study indicates centrin protein may not have a role in the cellular movement.

Regarding the *centrin* gene expression level in the logarithmic phase *L. infantum* promastigote and amastigotes, although no significant overexpression was found, the results of this study revealed that the mRNA of *L. infantum centrin* gene is slightly more expressed in amastigotes than logarithmic promastigotes. This finding is somewhat consistent with the results of Selvapandiyan et al. [23], where they report centrin protein is equally expressed in the promastigote and amastigote forms of *L. donovani*. The results of the mentioned study have shown a defect in cell division among amastigote forms of *L. donovani centrin* gene knockout (*LdCen*^*−/−*^) while promastigote forms of *LdCen*^*−/−*^ proliferate without any defect. It seems other types of proteins, such as calmodulin, γ-tubulin, and other types of centrin proteins, are responsible for basal body duplication in the promastigote forms. The lack of significant difference in the *centrin* expression between non-motile amastigote and promastigote forms confirms the fact that *centrin* has no role in parasite movement.

*Leishmania* parasites, especially the zoonotic species, have a complex transmission dynamic that is dependent on environmental conditions, the distribution and biology of the vector, the reservoirs involved, and the health, social, and economic aspects that affect the human host [25]. The strategies and interventions for the control of VL, including control of vectors and reservoirs and the early detection and treatment of human cases, faced some economic and moral limitations [26]. The mentioned complex scenario for the transmission dynamics of *L. infantum*, on the other hand, makes it even more difficult to establish effective control measures and highlights the clear need for the development of an effective prophylactic vaccine against CanL [27].

Our findings, besides the results obtained by Selvapandiyan et al. [15, 23], showed a significant dependence of the *Leishmania* parasite’s amastigote forms on the *centrin* gene activity. *Centrin* is a single copy gene [15], and its protein product as one of the main proteins required for duplication of the basal body and cell division could be an appropriate target for the prevention of *L. infantum* amastigotes proliferation in host cells. Defects in basal body duplication and cytokinesis in the *L. infantum* amastigote stage seem to be an attractive way to reach a genetically modified live attenuated vaccine against CanL.

In this study, because of financial constraints, we just collected three *L. infantum* isolates from various endemic areas of Iran. Indeed, by evaluating more isolates, a more precise picture of the *centrin* expression profile could be achieved.

## Conclusions

Analysis of the *centrin* gene expression in Iranian isolates of *L. infantum* showed this gene is more expressed in exponential than stationary phases, which highlights the potential role of *centrin* in the proliferation of these parasites. Understanding the role of *centrin* in *L. infantum* proliferation is a cornerstone to alter the survival of the parasite in the vertebrate host and could be a promising clue to the development of a genetically modified live attenuated vaccine against CanL.

## Methods

### Isolates and culture

We used three *Leishmania* parasites isolated from domestic dogs in endemic areas of CanL in Iran. The parasites were grown in RPMI 1640 (Gibco, Germany) culture medium supplemented with 20 % heat-inactivated fetal bovine serum (FBS) (Gibco, Germany), plus penicillin (100 U/ml) and streptomycin (100 µg/mL) (Sigma, USA), at pH 7.4 and incubation temperature of 25°C [28].

### Molecular characterization

#### DNA extraction

We harvested 2 mL well-grown promastigotes by centrifugation (800 g, 5 min at 4°C) and washed twice using sterile phosphate-buffered saline (PBS). Total DNA was extracted from the parasites using AccuPrep® Genomic DNA Extraction Kit (Bioneer, Korea) according to the manufacturer’s instructions and stored at -20°C until the subsequent process.

#### PCR-RFLP

PCR-RFLP performed using the NAGT gene, which amplified using the primer set, forward (5’-TCATGACTCTTGGCCTGGTAG-3’) and reverse (5’-CTCTAGCGCACTTCATCGTAG-3’) [29] which amplifies a fragment of approximately 1450-60 base-pair (bp) in *Leishmania* parasites (Fig. 2 Lane 2). We carried out the PCR amplification in a final reaction mixture containing 25 µL including 12.5 µL 2x red PCR Master Mix (Ampliqon, Denmark), 1 µL of each primer (10 pmol), 1 µL of the extracted DNA and 9.5 µL of sterile distilled water. We applied a negative control in each run. PCR was performed in the thermal cycler (PeqLab, Germany) using the following cycling protocol: an initial denaturation step at 94°C for 4 min, followed by 30 cycles of denaturation at 94°C for 1 min, annealing at 53°C for 1 min and extension at 72°C for 1 min, followed by a final extension step at 72°C for 8 min.

Species identification was carried out using RFLP analysis. Acc1 enzyme (Thermo Scientific, USA) can provide different digestion patterns in different *Leishmania* species. For this purpose, we added 10 µL of the amplicon, 2 µL of the enzyme buffer, 1 µL of the Acc1 enzyme and 17 µL distilled water to the reaction. The mixture was incubated at 37°C for 12 h and then visualized on 2 % agarose gel [29].

We used pre-confirmed *L. infantum*, *Leishmania major*, and *Leishmania tropica* species by PCR-RFLP [30], available at the School of Public Health, Tehran University of Medical Sciences as positive controls (Fig. 1 Lane 3–5, respectively).

### ***Centrin *gene expression**

#### Cell culture and amastigote isolation

Mouse macrophage cell line RAW264.7 (ATCC number TIB-71) was obtained from the Iranian Biological Resource Center, Tehran, Iran. The macrophage cells were cultured in RPMI 1640 medium supplemented with 20 % heat-inactivated FBS, penicillin (100 U/mL), and streptomycin (100 µg/mL) at 37°C in a humidified atmosphere of 5 % CO_2_ in 25 cm^2^ culture flasks (SPL, Korea). Following 72 h incubation (60 to 70 % confluency), we washed unattached macrophages off using the pre-warmed medium and incubated attached ones with the late stationary phase promastigotes (10 *Leishmania* per macrophage), as described by Mohtasebi et al. [31]. The culture was incubated in the same medium supplemented at 37°C for 4 to 6 h in the same condition until promastigotes were phagocyte by the macrophages. Then uninternalized parasites were washed off using pre-warmed medium.

Intracellular amastigotes were harvested and extracted from macrophages at 24 h and 72 h post-infection using a protocol as described by Decuypere et al. [32] with minor modifications. Briefly, 2 mL of 0.0125 % sodium dodecyl sulfate (SDS)/PBS were added to the culture flask and gently agitated until macrophages lifted and started to disintegrate. After mixing, the contents of the culture flask were aspirated through a 22-gauge needle, causing further shearing of the macrophages, and transferred to a 50 mL tube for differential centrifugation as follows; a 30 g centrifugation to separate the macrophages and a 700 g centrifugation to obtain the amastigote pellet.

#### RNA extraction and cDNA synthesis

Total RNA was obtained from the 5 × 10^6^ logarithmic and stationary phase promastigotes (96 h and 192 h post-subculture, respectively) and also from the 5 × 10^6^ axenic amastigotes at 24 h and 72 h post-infection of three *L. infantum* isolates, using FavorPrep™ Total RNA Extraction Mini Kit (Favorgen, Taiwan), following the manufacturer’s instructions. We performed an agarose gel electrophoresis to assess the overall quality of total RNA. The quantity of total RNA was also estimated using NanoDrop™ One/One^C^ Microvolume UV-Vis Spectrophotometer (Thermo Scientific, USA). Samples having an A_260_/A_280_ ratio of between 1.8 and 2.0 were considered. To avoid any genomic DNA contamination, we treated the extracted RNA using DNase (Qiagen, Germany) following the manufacturer’s recommendations. Total RNA was reverse transcribed from 1 µg of total RNA using the cDNA Synthesis Kit (YTA, Iran) according to the manufacturer’s instructions. Afterward, we evaluated the integrity of the synthesized cDNA through amplification of the *α-tubulin* as a housekeeping gene using primer set, forward (5’-CAGGTGGTGTCGTCTCTGAC-3’) and reverse (5’-TAGCTCGTCAGCACGAAGTG-3’), under the condition as described previously [28]. The PCR result was considered positive when a single band of 119 bp was observed.

#### Real‐time RT-PCR

We conducted a real-time RT-PCR assay to analyze the expression level of the *centrin* gene. *Centrin* primers were obtained using the Primer-BLAST tool [33] including forward (5’-CCGCTCTATGCACACAGACT-3’) and reverse (5’- AGGTCGAAGAGCTGAAAGGC-3’) which amplifies a 126 bp fragment in the experiments. The housekeeping gene, *α-tubulin*, was used as endogenous control and run along with the gene tested. RT-PCR was performed in duplicate with 20 µL volumes containing 10 µL RealQ Plus 2x Master Mix Green (Ampliqon, Denmark), 1 µL cDNA, 1 µL of each primer (10 pmol), and 7 µL distilled water in a StepOne™ Real-time PCR System (Applied Biosystems, USA). The PCR condition was as follows: initial denaturation at 95°C for 30 sec, 40 cycles of 10 sec at 95°C and 30 sec at 60°C followed by a melt curve analysis using temperature increments of 0.3°C every 30 sec to ascertain amplification of the expected product. A negative control, consisting of non-template water instead of cDNA, was used in each run of real-time RT-PCR.

#### Data analysis

The relative expression of the *centrin* gene in the *L. infantum* isolates was estimated and normalized to the internal control gene (*α-tubulin*) using the 2^−ΔΔct^ method [34]. The significance of differences was determined by the relative expression software tool (REST, ). All experiments conducted in duplicate and the obtained results have presented as the mean ± standard deviations. The expression ratio results of the target gene were tested for significance by a Pair Wise Fixed Reallocation Randomization Test© and plotted using standard error estimation via a complex Taylor algorithm, calculated by REST. The samples with a *p-*value of < 0.05 were considered significantly different among the groups [35].

## Data Availability

The datasets generated and analyzed during the current study may be made available from the corresponding author on request.

## Abbreviations

CanL: Canine leishmaniasis
VL: Visceral leishmaniasis
PCR: Polymerase chain reaction
bp: Base-pair
RFLP: Restriction fragment length polymorphism
DNase: Deoxyribonuclease
RT: Reverse transcription
SDS: Sodium dodecyl sulfate
PBS: Phosphate-buffered saline
FBS: Fetal bovine serum
NAGT: N-acetylglucosamine-1-phosphate transferase
Acc1: Acetyl-coenzyme A carboxylase 1
cDNA: Complementary DNA
*LdCen*^*−/−*^: *Leishmania donovani centrin* gene knockout
mRNA: Messenger RNA
BLAST: Basic local alignment search tool
REST: Relative expression software tool
